# Bacterial chitin degradation—mechanisms and ecophysiological strategies

**DOI:** 10.3389/fmicb.2013.00149

**Published:** 2013-06-14

**Authors:** Sara Beier, Stefan Bertilsson

**Affiliations:** ^1^Department of Ecology and Genetics, Limnology, Uppsala UniversityUppsala, Sweden; ^2^Laboratoire d'Océanographie Microbienne, Observatoire Océanologique, UPMC Paris 06, UMR 7621Banyuls sur mer, France; ^3^Laboratoire d'Océanographie Microbienne, Observatoire Océanologique Centre National de la Recherche Scientifique, UMR 7621Banyuls sur mer, France

**Keywords:** chitin, particles, organic matter, bacteria, interactions, cross-feeding, glycoside hydrolase

## Abstract

Chitin is one the most abundant polymers in nature and interacts with both carbon and nitrogen cycles. Processes controlling chitin degradation are summarized in reviews published some 20 years ago, but the recent use of culture-independent molecular methods has led to a revised understanding of the ecology and biochemistry of this process and the organisms involved. This review summarizes different mechanisms and the principal steps involved in chitin degradation at a molecular level while also discussing the coupling of community composition to measured chitin hydrolysis activities and substrate uptake. Ecological consequences are then highlighted and discussed with a focus on the cross feeding associated with the different habitats that arise because of the need for extracellular hydrolysis of the chitin polymer prior to metabolic use. Principal environmental drivers of chitin degradation are identified which are likely to influence both community composition of chitin degrading bacteria and measured chitin hydrolysis activities.

## Introduction

The occurrence of chitin is widespread in nature and chitin serves as a structural element in many organisms, e.g., fungi, crustaceans, insects or algae (Gooday, 1990a,b). Chitin is composed of linked amino sugar subunits. Similar to cellulose and murein, it makes a shortlist of highly abundant biopolymers with enormous global production rates estimated at approximately 10^10^–10^11^ tons year^−1^ (Gooday, 1990a; Whitman et al., 1998; Kaiser and Benner, 2008). There are no reports of quantitatively significant long-term accumulation of chitin in nature, implying efficient degradation and turnover (Tracey, 1957; Gooday, 1990a).

In accordance with the abundance and ubiquity of chitin, chitin-degrading enzymes are also detected in many types of organisms, such as fungi, bacteria (Gooday, 1990a), archaea (Huber et al., 1995; Tanaka et al., 1999; Gao et al., 2003), rotifers (Štrojsová and Vrba, 2005), some algae (Vrba et al., 1996; Štrojsová and Dyhrman, 2008), but also carnivorous plants or in digestional tracts of higher animals (Gooday, 1990a).

Bacteria are believed to be major mediators of chitin degradation in nature. In soil systems, chitin hydrolysis rates have been shown to correlate with bacterial abundance (Kielak et al., 2013), but depending on temperature, pH, or the successional stage of the degradation process, also fungi may be quantitatively important agents of chitin degradation (Gooday, 1990a; Hallmann et al., 1999; Manucharova et al., 2011). In aquatic systems, plating and *in situ* colonization experiments convincingly demonstrates that bacteria are the main mediators of chitin degradation (Aumen, 1980; Gooday, 1990a). However, occasionally, dense fungal colonization of chitinous zooplankton carapaces has been observed (Wurzbacher et al., 2010) and some diatoms have also been shown to hydrolyze chitin oligomers (Vrba et al., 1996, 1997). A further source of chitin modifying enzymes in aquatic systems are enzymes released during molting of planktonic crustaceans (Vrba and Machacek, 1994). Nevertheless, it is not yet clear whether the enzymes released by diatoms and molting zooplankton react with particulate chitin to any significant extent or if their hydrolytic activity is limited to dissolved chitin oligomers.

Chitin is the polymer of (1→4)-β-linked N-acetyl-D-glucosamine (GlcNAc). The single sugar units are rotated 180° to each other with the disaccharide N,N′-diacetylchitobiose [(GlcNAc)_2_] as the structural subunit. In nature, chitin varies in the degree of deacetylation and therefore the distinction from chitosan, which is the completely deacetylated form of the polymer, is not strict. Chitin is classified into three different crystalline forms: the α-, β-, and γ-form, which differ in the orientation of chitin micro-fibrils. With few exceptions, natural chitin occurs associated to other structural polymers such as proteins or glucans, which often contribute more than 50% of the mass in chitin-containing tissue (Attwood and Zola, 1967; Schaefer et al., 1987; Merzendorfer and Zimoch, 2003). Chitin is a structural homologue of cellulose where the latter is composed of glucose instead of GlcNAc subunits. Also murein in bacterial cell walls can be considered a structural chitin homologue, as it is composed of alternating (1→4)-β-linked GlcNAc and N-acetylmuramic acid units.

**Figure 1:**
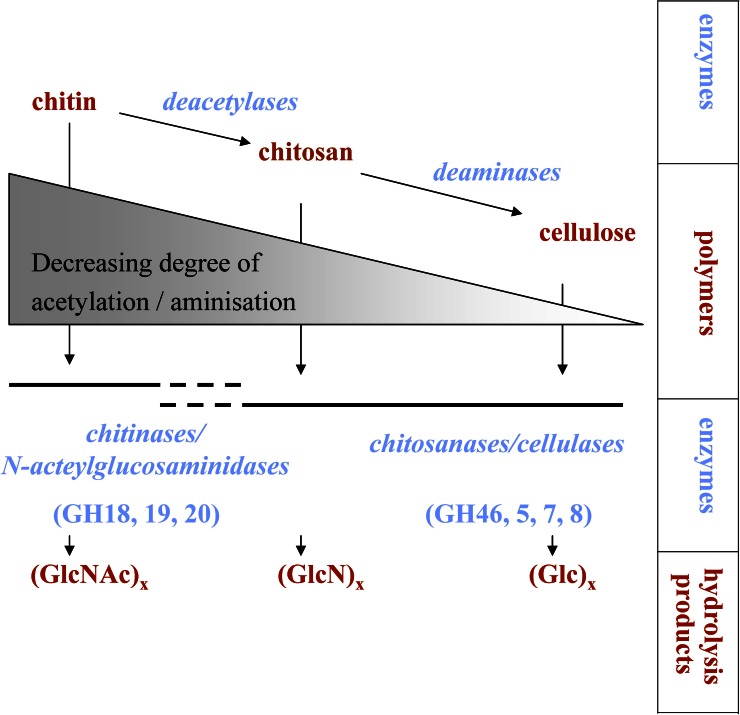
**Processes involved in chitin degradation.** If deacetylation and deamination processes are very active, chitosan or possibly even cellulose-like molecules might be produced. GH, glycoside hydrolase family; GlcNAc, N-acetylglucosamine; GlcN, glucosamine; Glc, glucose.

A process is called chitinoclastic if chitin is degraded. If this degradation involves the initial hydrolysis of the (1→4)-β-glycoside bond, as seen for chitinase-catalyzed chitin degradation, the process is called chitinolytic. Growth on chitin is not necessarily accompanied by the direct dissolution of its polymeric structure. Alternatively, chitin can be deacetylated to chitosan or possibly even cellulose-like forms, if it is further subjected to deamination (Figure 1). Such a degradation mechanism has been suggested in some early studies (ZoBell and Rittenberg, 1938; Campbell and Williams, 1951). Chitinases and chitosanases overlap in substrate specificity, while their respective efficiency is controlled by the degree of deacetylation of the polymeric substrate (Somashekar and Joseph, 1996) (Figure 1). Besides specific chitosanases, also cellulases can possess considerable chitosan-cleaving activity (Xia et al., 2008). Furthermore, lysozyme has also been shown to hydrolyze chitin, even if processivity is low when compared to true chitinases (Skujiņš et al., 1973). Cellulases can also bind directly to chitin (Ekborg et al., 2007; Li and Wilson, 2008), but there are no reports of these enzymes actually hydrolyzing the polymers.

Few studies have compared the quantitative importance of different chitinoclastic pathways, and the studies available suggest that chitin degradation via initial deacetylation might be more important in soil and sediment compared to water environments (Hillman et al., 1989; Gooday, 1990a). The quantitative importance of different chitinoclastic pathways from a global perspective has, to the best of our knowledge, never been assessed. In the following sections, we will focus on the chitinolytic pathway.

**Figure 2:**
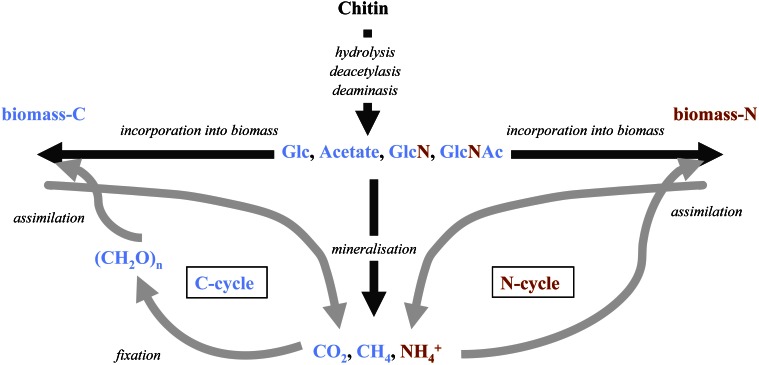
**Fate of possible chitin degradation intermediates and degradation products at the interface of the global N and C-cycles: during the first degradation steps chitin is cleaved into small organic molecules that can directly be reintegrated into cell material or mineralized and potentially removed from the system.** GlcNAc, N-acetylglucosamine; GlcN, glucosamine; Glc: glucose.

The quantitative significance of chitin has been recognized for some time and there has been great interest in identifying processes and factors controlling its degradation. Accordingly, the biochemistry, molecular biology, and biogeochemistry of chitin degradation have been summarized in reviews published already some 20 years ago (Gooday, 1990a; Cohen-Kupiec and Chet, 1998; Keyhani and Roseman, 1999). More recently, the development and widespread use of culture-independent molecular methods in microbial ecology have enabled further dissection of microbial processes controlling chitin degradation in more complex natural environments and diverse microbial communities. These methodological advances combined with the significance of chitin as a critical link between the carbon and nitrogen cycles (Figure 2) has led to a revived interest in the quantitative importance of chitin turnover in marine systems (Souza et al., 2011).

There is clearly a need for an updated account of the diverse mechanisms involved in chitinolysis and the ecological consequences of this process for bacteria. A focus on bacteria rather than all other organisms involved in chitin degradation is warranted since bacterial chitin degradation takes place in all major ecosystems and because their metabolism and growth have such a central role in most ecosystem-scale biogeochemical cycles. However, also non-bacterial or non-chitinolytic chitin-degraders will occasionally be mentioned and discussed where their activities would influence bacterial chitin degradation. In light of recent developments in molecular methods, a particular emphasis will be on how the participation and interactions of specific microbial populations and community composition influence the process. We further identify gaps in knowledge and needs for further research.

## Biochemistry of chitin hydrolysis

Chitin degradation is a highly regulated process, and the hydrolytic enzymes are induced by products of the chitin hydrolyses, GlcNAc (Techkarnjanaruk et al., 1997), or soluble chitin oligomers (GlcNAc)_2–6_ (Keyhani and Roseman, 1996; Miyashita et al., 2000; Li and Roseman, 2004; Meibom et al., 2004), depending on the organism under scrutiny. In contrast to (GlcNAc)_2_, GlcNAc has also been reported to act as a suppressor of chitinase expression in a *Streptomyces* strain (Miyashita et al., 2000) and this may be because its main origin in natural systems could be from murein in cell walls rather than chitin (Benner and Kaiser, 2003). Other factors more generally regulating the expression of these and other hydrolytic enzymes are nutrient regime and availability of other, more readily available growth substrates (Techkarnjanaruk et al., 1997; Keyhani and Roseman, 1999; Delpin and Goodman, 2009a,b). The variety of regulating factors are likely to reflect the wide range of ecological niches occupied by chitin degraders.

Complete lysis of the insoluble chitin polymer typically consists of three principal steps (1) cleaving the polymer into water-soluble oligomers, (2) splitting of these oligomers into dimers, and (3) cleavage of the dimers into monomers. The first two steps are usually catalyzed by chitinases. The occurrence of chitinases in bacteria is widespread among phyla and the production of multiple chitinolytic enzymes by individual bacterial strains appear to be a common trait (e.g., Fuchs et al., 1986; Romaguera et al., 1992; Saito et al., 1999; Shimosaka et al., 2001; Tsujibo et al., 2003). Chitinases are typically grouped into family 18 and 19 glycoside hydrolases. The latter are rare in bacteria except for some members of the genus *Streptomyces* (Ohno et al., 1996; Saito et al., 1999; Watanabe et al., 1999; Shimosaka et al., 2001; Tsujibo et al., 2003). It has been hypothesized that family 18 and 19 glycoside hydrolases have evolved separately, as genes belonging to these two analogous gene families show little or no sequence homology, nor share the same molecular-level catalytic mechanism (Perrakis et al., 1994; Davies and Henrissat, 1995; Hart et al., 1995). The occurrence of multiple genes in a single organism may be the result of gene duplication or acquisition of genes from other organisms via lateral gene transfer (Hunt et al., 2008). In support of the former mechanism, different chitinase gene sequences found within single organisms are often almost identical. However, there are examples where chitinase genes coexisting in a single organism are very different and cluster with chitinase sequences from rather distantly related organisms (Saito et al., 1999; Suzuki et al., 1999; Karlsson and Stenlid, 2009). This suggests lateral gene transfer also between distantly related organisms.

Multiple chitinases within a single organisms are believed to lead to a more efficient use of the respective substrate as a result of synergistic enzyme interactions or contrasting affinities to different substrate forms (Svitil et al., 1997). One example of this is the extensively studied chitinase system of *Serratia marcescens*, which is based on several chitinases with slightly different functions. *S. marcescens* produces four family 18 chitinases ChiA, ChiB, ChiC1, and ChiC2, all of which are released into the surrounding medium (Suzuki et al., 1998). ChiC2 results from a posttranslational modification of ChiC1 (Gal et al., 1998; Suzuki et al., 1999) and hydrolytic activities of ChiC2 were lower on crystalline substrates compared to ChiC1 whereas no further differences were identified (Suzuki et al., 1999), leaving the function of ChiC2 unclear. By combining ChiA, ChiB, and ChiC1, synergistic effects on chitin degradation have been observed, implying differential action sites and/or molecular reaction mechanisms for the three enzymes (Suzuki et al., 2002). Indeed it was later shown that ChiC is a non-processive endoenzyme that cleaves the chitin polymer randomly, whereas both ChiA and ChiB are processive enzymes cleaving off disaccharides while sliding along the chitin polymer (Horn et al., 2006; Sikorski et al., 2006). Multiple action mechanisms are also implied for each of the latter two chitinases as it has been demonstrated that ChiA and ChiB degrade β-chitin microfibrils unidirectionally from opposite ends of the polymer (Hult et al., 2005). Still, the major end products from all three enzymes are disaccharides, whereas monosaccharides are produced as byproducts in substantially lower amounts (Horn et al., 2006). There are other examples where multiple enzymes within an organism catalyze the metabolism of a single substrate, with cellulose as a pertinent example (Rabinovich et al., 2002). It seems conceivable that enzyme multiplicity might be a general feature in polymer degrading processes caused by the structural complexity of the substrate. This would then allow parallelized or successive contrasting modes of action on the same polymer.

β-N-acetyl-hexosaminidases, usually affiliated with family 20 glycoside hydrolases, finally cleave GlcNAc from the non-reducing end of the water soluble chitin oligomers produced by chitinases (Scigelova and Crout, 1999). In bacteria, this last step typically takes place in the cytoplasm or the periplasmic space (Bassler et al., 1991; Keyhani and Roseman, 1996; Drouillard et al., 1997; Techkarnjanaruk and Goodman, 1999). In some bacteria, enzymes other than the family 20 glycoside hydrolases are involved in hydrolyzing GlcNAc from chitin oligomers (Tsujibo et al., 1994; Chitlaru and Roseman, 1996; Park et al., 2000). Recent research also suggests that some family 20 glycoside hydrolases can cleave GlcNAc directly from chitin polymers and hence function as chitinases (LeCleir et al., 2007).

Chitin degradation is also influenced by more cryptic factors. For example, a chitin-binding protein without any catalytic domain has been shown to facilitate the degradation of β-chitin by disrupting the crystalline chitin polymer structure (Vaaje-Kolstad et al., 2005). The protein showed significant sequence similarity to a gene product in *Streptomyces olivaceoviridis* known to have high affinity to α-chitin (Schnellmann et al., 1994). It has been proposed that the ability to produce such proteins with high specific affinity to a certain crystalline chitin structure may be decisive for the ability of bacteria to differentiate and react to specific crystalline chitin structures (Svitil et al., 1997). Such chitin-binding domains may also influence chitin degradation indirectly by facilitating adhesion of cells to chitinous substrates, a trait that is of particular importance in aquatic environments (Montgomery and Kirchman, 1993, 1994; Pruzzo et al., 1996).

Since the insoluble chitin polymer has to be cleaved outside of the bacterial cell barrier, metabolic use of chitin also relies on efficient uptake systems for hydrolysis products. In some cultivated bacterial strains, PTS (phosphoenolpyruvat: glycose phosphotransferase system) transporters are responsible for the main GlcNAc uptake. However, the uptake activity of other specific GlcNAc transporters as well as transporters with a broader substrate range (including sugar monomers like glucose, glucosamine, fructose and mannose) have also been described (Mobley et al., 1982; Postma et al., 1993; Bouma and Roseman, 1996). The quantitative importance of these two substrate uptake strategies, highly specific or more versatile, is not clear and culture independent assays based on inhibition experiments provide contrasting results concerning the specificity of GlcNAc-uptake systems. Whereas Riemann and Azam (2002) found a specific inhibition of the bacterial GlcNAc-uptake by glucose, this was not the case in an earlier study by Vrba et al. (1992). Reasons for such conflicting results could be a different set of organisms being present at the respective sampling sites, i.e., due to the different environment under scrutiny in the respective study (marine vs. freshwater) or seasonal differences in nutrient status of the system.

Radiotracer studies in lake water suggest differentiation in GlcNAc and (GlcNAc)_2_ uptake among phylogenetic groups of bacteria with the (GlcNAc)_2_ uptake being quantitatively more important (Beier and Bertilsson, 2011). This implies that the two hydrolysis products are taken up by different transporter systems in freshwater ecosystems. The earlier discussed role of (GlcNAc)_2_ as main hydrolysis product of chitinases (Horn et al., 2006) and the quantitative importance of the (GlcNAc)_2_ uptake mentioned above (Beier and Bertilsson, 2011) corroborates the observation that bacterial β-N-acetyl-hexosaminidases are often intracellular enzymes. Consequently, the relevance of (GlcNAc)_2_ transport through the cell barrier during the process of chitin degradation is evident.

## Species interactions during chitin degradation in different habitats

Particles that contain chitin can act as a source of chitin degradation intermediates to the surrounding medium (Smith et al., 1992; Kirchman and White, 1999). This implies that chitinolytic bacteria sometimes process more chitin polymers than they are able to use themselves. For instance, only a minority of cells in a pure culture of *Pseudoalteromonas* S91 growing on chitin as a sole source of carbon and nitrogen hydrolyzed chitin (Baty et al., 2000a,b). It was assumed that cells with no apparent chitinase activity fed on hydrolysis products produced in excess by the chitinase-positive subpopulation. This type of multicellular cooperation is a strategy often observed in bacteria (Shapiro, 1998) and has been described for several chitinolytic strains (Gaffney et al., 1994; Chernin et al., 1998; DeAngelis et al., 2008). Considering the complexity of the chitinolytic cascade, with approximately 50 different proteins being induced (Keyhani and Roseman, 1999; Li and Roseman, 2004; Meibom et al., 2004), a partitioning of the clonal population into a chitinase up-regulated subpopulation that supply hydrolysis products to their kin could be a successful survival mechanism. Such intraspecific cross-feeding might also explain the excess enzymatic activity observed on particles in aquatic systems (Smith et al., 1992; Kirchman and White, 1999). However, in natural environments, the release of hydrolysis products would not only serve specific clonal populations, but also open up the possibility for interspecific cross-feeding. The existence of interspecies cross-feeding therefore seems plausible and studies on bacterial pure cultures have indeed demonstrated that there are organisms that grow on GlcNAc (Kaneko and Colwell, 1978) or (GlcNAc)_2_ (Keyhani and Roseman, 1997) without possession of the enzymes for chitinolytic activity.

The habitat structure in which polymer degradation takes place might have great consequences for this kind of interspecies interactions. The flux of dissolved substances as hydrolyses products is physically constrained in aerated soils. Accordingly hydrolyses products will remain in close spatial proximity to the place of enzymatic action. In terrestrial systems, interspecies metabolic interactions will therefore likely be limited to organisms growing directly adjacent to each other in biofilms. Besides commensal sharing of such hydrolysis products (Everuss et al., 2008) there is also a potential for specialized interactions between organisms such as synergistic coupling and the recently described parasitism that rely on bi-directional exchange of e.g., metabolic inhibitors and chitin degradation intermediates among specific bacterial populations (Jagmann et al., 2010). In contrast, released hydrolyses products in aquatic systems will be subject to transport by diffusion and hydrological flow away from the site where hydrolysis took place. Because of the facilitated transport of hydrolysis products away from the hydrolytic site, quantitatively significant cross-feeding events can occur over longer distances in this biome as observed previously (Cho and Azam, 1988; Beier and Bertilsson, 2011; Eckert et al., 2013). Thus, it seems likely that such long-distance cross-feeding relationships could favor rather unspecific and unidirectional commensal interactions, where the receiving organism is less likely to critically depend on the interaction. Sediments or waterlogged soils in wetlands may represent habitats with intermediate transport constraints, locally sharing transport characteristics with both environments outlined above.

To the best of our knowledge, no studies exist that target species interactions during chitin degradation in soil environments specifically, nor are we aware of studies that compare the above suggested general differences in cross-feeding between aquatic and terrestrial habitats. However, a number of culture-independent studies in aquatic environments that quantify the fraction of chitin degraders vs. chitin consumers in the total bacterial community support the existence of significant cross-feeding during chitin degradation (Table 1): chitinolytic organisms were estimated to represent 0.1–5.8% (average about 1%) of all prokaryotes in a variety of aquatic ecosystems (Cottrell et al., 1999; Beier et al., 2011). An even lower fraction of cells displayed active chitinolytic activity in natural aquatic habitats (0–1.9%) (Beier and Bertilsson, 2011; Beier et al., 2012). In contrast, between 4 and 40% of the bacteria, or one third of the DNA-replicating bacteria, were shown to incorporate chitin hydrolysis products (Nedoma et al., 1994; Riemann and Azam, 2002; Beier and Bertilsson, 2011; Eckert et al., 2013).

**Table 1:** **Fraction of chitinolytic, chitinolytically active, and chitin hydrolysis products incorporating cells (no results of culture-dependent studies are listed here, since quantitative values are likely strongly biased)**.

**Fraction of cells**	**System**	**Method**	**References**
5.5%	Brackish water	Chitinase genes in metagenomes (fraction of chitinolytic cells)	Cottrell et al., 1999
0.1%	Marine water	Chitinase genes in metagenomes (fraction of chitinolytic cells)	Cottrell et al., 1999
3.1%	Freshwater	Chitinase genes in metagenomes (fraction of chitinolytic cells)	Beier et al., 2011
0.7–1.5%	Brackish water	Chitinase genes in metagenomes (fraction of chitinolytic cells)	Beier et al., 2011
0.2–5.8%	Marine water	Chitinase genes in metagenomes (fraction of chitinolytic cells)	Beier et al., 2011
1.3%	Hypersaline water	Chitinase genes in metagenomes (fraction of chitinolytic cells)	Beier et al., 2011
Not detectable	Freshwater	^1^ELF® 97 (fraction of chitinolytically active cells)	Beier and Bertilsson, 2011
up to 1.9%	Freshwater	ELF® 97 (fraction of chitinolytically active cells)	Beier et al., 2012
4.2–38.9%	Freshwater	^2^MAR-FISH (fraction of GlcNAc incorporating cells)	Nedoma et al., 1994
7%	Freshwater	MAR-FISH (fraction of (GlcNAc)_2_ incorporating cells)	Beier and Bertilsson, 2011
6–7%	Freshwater	MAR-FISH (fraction of GlcNAc incorporating cells)	Beier and Bertilsson, 2011
8%	Freshwater	MAR-FISH (fraction of GlcNAc incorporating cells)	Eckert et al., 2013
43% of DNA synthesizing bacteria	Marine water	Streptozotocin sensitivity (fraction of GlcNAc incorporating cells)	Riemann and Azam, 2002

1 ELF® 97: ELF® 97 chitinase-N-acetylglucosaminidase substrat.

2 MAR-FISH: microautoradiography—fluorescence in situ hybridization.

The assumption that the uptake of polymer-derived metabolites in aquatic system often occurs over longer distances is supported by the observation that typically free-living bacterial groups appear to be quantitatively important receivers of this hydrolyzed material (Cho and Azam, 1988; Beier and Bertilsson, 2011; Eckert et al., 2013). For such long-distance substrate acquisition, the free-living organisms receiving the hydrolysis products are likely to profit from the action of other hydrolytic bacteria that are in close proximity to the polymeric substrate: any hydrolytic enzymes produced by free living cells across such long distances would have a low probability of encountering the substrate and even in this case the majority of resulting hydrolysis products would not be encountered by the free-living cell. Model findings indicate that the area around a polymer-hydrolyzing bacterium, from which hydrolysate can be efficiently collected, is limited to approximately 10 μm distance from the polymeric source (Vetter et al., 1998). Free-living bacteria might occasionally be within this distance to a chitinous particle, but it is uncertain whether the gain from such occasional degradation product uptake can balance the costs for maintenance of the polymer hydrolyzing machinery. On the other hand, it has recently been demonstrated that a member of the typically free-living lineage *Actinobacteria* ac1 hosts genes to take up GlcNAc while also encoding a chitinase gene (Garcia et al., 2013). However, it still remains to be demonstrated, whether or not these gene products can solubilize polymeric chitin.

Because of the more pronounced dilution of the released hydrolyses products in aquatic systems, a successful receiving organism residing such a long distance from the polymer hydrolysis site would likely also feature high affinity uptake systems. In agreement with this idea, Boyer (1994) observed radiolabeled chitin degradation intermediates in sediment but not in water after incubating both type of samples with ^14^C labeled chitin. This suggests that organisms with higher substrate affinity are present in the water samples compared to organisms present in the sediment. It remains to be tested whether the remaining intermediates in sediments would be metabolized over longer timescales or become resistant to further degradation by diagenetic processes. It is also unknown, whether organisms with high substrate affinity influence the efficiency of polymer degradation or if they are irrelevant for the overall ecosystem functioning.

## Taxonomic identity of chitinoclastic organisms

Qualitative characterization of the chitinolytic community by means of culture-independent molecular methods such as PCR amplification of chitinase genes or metagenomic approaches usually results in a rather rough level of identification. This is due to the supposedly extensive lateral gene-transfer and the limited taxonomic coverage of characterized reference organisms. One consequence of this is that a large number of chitinase gene sequences cannot be clearly affiliated to specific taxa. However, at a broader phylogenetic resolution recent studies in aquatic environments indicate that group A chitinases were by far the most abundant phylogenetic subgroup of family 18 glycoside hydrolases (Beier et al., 2011). More detailed information about the taxonomic identity of microorganisms that consume the chitin degradation products can be obtained by either cultivation approaches or by using radiotracer techniques. The bias inherent in studies that describe natural bacterial communities using exclusively cultivation-dependent approaches are well-known (Amann et al., 1995), but the bias appear to be of quantitative rather than qualitative concern.

In aquatic systems, *Cytophaga-Flavobacteria* are known to profit from chitin addition and have been detected in dense cluster on chitinous particles where they also assimilate chitin hydrolysis products (Cottrell and Kirchman, 2000; Beier and Bertilsson, 2011). This suggests a central role of *Cytophaga-Flavobacteria* in aquatic chitin degradation where they also benefit from this material as a substrate. In contrast, in soil environments bacteria affiliated with *Actinomyces* are often identified as being active chitin degraders, as they display enhanced growth and activity upon chitin addition. Members of this phylum are also frequently recovered in cultivation dependent studies of chitin degraders (Metcalfe et al., 2002; Manucharova et al., 2011). However, in both of these biomes, chitinoclastic bacteria from other phylogenetic groups, including *Proteobacteria* and *Firmicutes*, are also commonly observed (Cottrell et al., 2000; Brzezinska and Donderski, 2006; Yasir et al., 2009). The high phylogenetic diversity within the frequently isolated chitinolytic bacteria may therefore reflect a high ecological diversity of chitin degraders and could also explain why chitin does not accumulate in nature, but instead seems to be degraded under all possible environmental conditions (Tracey, 1957; Gooday, 1990a).

The composition of the chitin utilizing community—including active degraders and organisms profiting from cross-feeding events—might be decisive for the fate of chitin. It seems plausible that i.e., gram-positive chitin consumers use a higher percentage of GlcNAc in anabolic processes to synthesize the murein needed in abundance for production of their cell wall, while gram-negative bacteria might allocate more of these substrates to catabolic energy acquisition. Indeed, the fraction of hydrolyzed chitin respired to CO_2_ in natural ecosystems varies considerably between 30 and 93% (Table 2). Whereas the presence of other substrates has been shown to influence mineralization rate of GlcNAc (Mobley et al., 1982), it remains to be determined if the species composition of chitin consumers, as speculated above, has any significant influence of the actual chitin mineralization rates.

**Table 2:** **Fraction of hydrolyzed chitin that is mineralized**.

**Chitin mineralization (% of hydrolyzed chitin)**	**System**	**Method**	**References**
93%	Freshwater	^1 14^C, 25°C, crab shells	Boyer, 1994
78%	Freshwater	^14^C, 15°C, crab shells	Boyer, 1994
30%	Brackish water	^14^C, purified fungal chitin	Kirchman and White, 1999
55–72%	Freshwater sediment	^14^C, 25°C, crab shells	Boyer, 1994
50–75%	Freshwater sediment	^14^C, 15°C, crab shells	Boyer, 1994

1 14 C: chitin mineralization estimated based on

14 C labeled tracer compounds.

Since the taxonomic identity of the chitin-degrading and chitin-utilizing organisms might be decisive to ecosystem functioning, i.e., as outlined above for mineralization rates of chitin, it seems important to learn more about key players involved in different environments. One feasible strategy might be to combine designed experiments with single-cell isotope tracer methods. Another option is the direct coupling of chitin degradation traits to other metabolic features and taxonomic affiliation via single cell genome sequencing of uncultured microorganisms (Stepanauskas and Sieracki, 2007).

## Dynamics of the chitinolytic community structure and chitin degradation rates

Chitin degradation is a regulated trait and chitin degraders will be able to also metabolize other substrates than chitin. Therefore, the coupling between the abundance and composition of the chitinolytic community and their collective hydrolytic activity might not always be strong. A number of different methods, such as weight loss, ^14^C labeled chitin tracer experiments or incubation experiments with colorimetric or fluorogenic substrate analogs, have been applied to measure hydrolytic activity during chitin degradation. Due to the variety of different methods applied, measuring i.e., potential or actual rates, individual values for chitin hydrolytic activity in different studies are difficult to compare directly (Tables 3, 4). Instead we will describe trends in environmental control of chitin degradation detected consistently across several studies and if possible compare these patterns to shifts in the chitinolytic community composition. All methods for activity measurements have in common that they do not differentiate between different organisms hydrolyzing the chitin. Depending on the method used, also enzymes other than chitinases, such as chitosanases, β-N-acetyl-hexosaminidases or lysozymes might contribute to the measured rates (Höltje, 1996; Vrba et al., 1996). Community shifts are in most cases detected by molecular analyses of group A chitinases of the family 18 glycoside hydrolase (Table 5). Bacterial as well as non-bacterial organisms capable of chitin hydrolysis, such as those that possess β-N-acetyl-hexosaminidases and lysozyme are also frequently carrying group A chitinase genes. The targeted group A chitinases can thus also include genes from fungi, algae and higher animals (Hobel et al., 2005; Beier et al., 2012). Therefore, most organisms that contribute to the measured chitinolytic process should be included in the community analyses.

**Table 3:** **Chitin hydrolysis rates measured in natural habitats (values from experimental manipulations measured along with controls from natural habitats were excluded from the table)**.

**Chitin hydrolysis rates**	**System**	**Method**	**References**
0.00043–0.0005% d^−1^	Marine water	^1 14^C, 1°C, synthesized chitin	Herwig et al., 1988
27% d^−1^	Freshwater	^14^C, 15°C, crab shells	Boyer, 1994
30% d^−1^	Freshwater	^14^C, 25°C, crab shells	Boyer, 1994
<1% d^−1^	Brackish water	^14^C, *in situ* T, purified fungal chitin	Kirchman and White, 1999
8.1% d^−1^	Brackish water	*In situ*—weight loss on squid pen—yearly mean	Gooday et al., 1991
0.5–4.4% d^−1^	Freshwater-sediment interface	*In situ*—weight loss on purified chitin—different seasons	Warnes and Rux, 1982
0.1–4.5% d^−1^	Brackish water-sediment interface	*In situ*—weight loss on squid pen—yearly mean	Gooday et al., 1991
12–16% d^−1^	Freshwater sediment	^14^C, 15°C, crab shells	Boyer, 1994
22–27% d^−1^	Freshwater sediment—sand	^14^C, 25°C, crab shells	Boyer, 1994
0.0002 – 0.005% d^−1^	Marine sediment	^14^C, 1°C, synthesized chitin	Herwig et al., 1988
2.6–2.8% d^−1^	Brackish sediment	*In situ*—weight loss on squid pen—yearly mean	Gooday et al., 1991
2.8% d^−1^	Brackish sediment	*In situ*—weight loss on squid pen—yearly mean	Gooday et al., 1991
1% d^−1^	Brackish sediment	Weight loss on squid pen	Hillman et al., 1989
^*^00.6–1.1% d^−1^	Soil	*In situ*—weight loss on crab shell chitin	Metcalfe et al., 2002

If possible, values given in different units in the original publications were transformed into a single unit.

1 14 C: degradation rates estimated based on

14 C labeled tracer compounds.

* Values derived from digitalized figures using the Engauge Digitizer Program (http://digitizer.sourceforge.net/index.php?c=5).

**Table 4:** **Chitinase and β-N-acetyl-hexosaminidase enzyme activities in natural habitats**.

**Enzyme activities**	**System**	**Method**	**References**
5.4 × 10^−5^ – 3.1 × 10^2^ nmol d^−1^ ml^−1^	Freshwater	^1^MUF-NAG, *in situ* T, 100 μM	Vrba et al., 1992
1.3 × 10^−5^ – 1.3 × 10^−4^ nmol d^−1^ ml^−1^	Freshwater	MUF-NAG, *in situ* T, 50 μ M	Beier et al., 2012
^*^2.8 × 10^2^ – 3.4 × 10^2^ nmol d^−1^ g^−1^ (wet)	Wetland sediment	^2^pNP-NAG, 25°C, 5 mM	Jackson and Vallaire, 2009
^*^4.2 × 10^−1^ – 1.4 × 10^2^ nmol d^−1^ ml^−1^ (wet)	Wetland sediment	MUF-NAG, respective annual mean T, 400 μ M	Kang et al., 2005
^*^1.7 × 10^0^ – 9.2 × 10^0^ μg d^−1^ g^−1^ (dry)	Saltmarsh sediment	pNP-NAG, 30°C, 5 mM	Duarte et al., 2008
^*^2.4 × 10^3^ – 7.1 × 10^3^ nmol d^−1^ g^−1^ (dry)	Soil	pNP-NAG, 25°C, 2 mM	Rietl and Jackson, 2012
Not detectable	Freshwater	^3^MUF-DC, 4°C, 50 μM	Köllner et al., 2012
Up to 5.4 × 10^1^ nmol d^−1^ ml^−1^	Freshwater	MUF-DC, *in situ* T, 50 μ M	Beier et al., 2012
4.2 × 10^−3^ – 2.1 × 10^−1^ nmol d^−1^ g^−1^ (dry)	Freshwater sediment	MUF-DC, 4°C, 50 μ M	Köllner et al., 2012
2.5 × 10^1^ – 7.5 × 10^3^ nmol d^−1^ g^−1^ (dry)	Soil	MUF-DC, 37°C, 60 μ M	Ueno et al., 1991
Up to 5.4 × 10^3^ nmol d^−1^ g^−1^ (dry)	Soil	^4^MUF-TC, 37°C, 25 μ M	Ueno et al., 1991
^*^5.4 × 10^3^ – 6.3 × 10^0^ μg d^−1^ g^−1^ (dry)	Brackish sediment	^5^DNP, 15°C, 0.5 mg ml^−1^	Hillman et al., 1989

If possible, values given in different units in the original publications were transformed into a single unit (listed values do not provide a complete overview on all measurements performed but display examples, values from experimental manipulations measured along with controls from natural habitats were excluded from the table).

1 MUF-NAG: β-N-acetyl-hexosaminidase/chitinase hydrolysis rates estimated based on the fluorogenic substrate analog N-acetyl-b-D-glucosaminide

2 pNP-NAG: N-acetyl-hexosaminidase/chitinase hydrolysis rates estimated based on the fluorogenic substrate analog pNP-β-N-acetylglucosaminide

3 MUF-DC: β-N-acetyl-hexosaminidase/chitinase hydrolysis rates estimated based on the fluorogenic substrate analog methylumbelliferyl-diacetyl-chitobioside

4 MUF-TC: β-N-acetyl-hexosaminidase/chitinase hydrolysis rates estimated based on the fluorogenic substrate analog methylumbelliferyl-diacetyl-chitotrioside

5 DNP: N-acetyl-hexosaminidase/chitinase hydrolysis rates estimated based on the fluorogenic substrate analog 3,4-dinitrophenyl-tetra-N-acetyl chitotetraoside

* Values derived from digitalized figures using the Engauge Digitizer Program (http://digitizer.sourceforge.net/index.php?c=5).

**Table 5:** **Chitinase gene copies numbers in natural habitats**.

**Gene copies**	**System**	**Method**	**References**
Up to 3.4 × 10^2^ ml^−1^	Freshwater	^1^qPCR on ^2^GH18 genes	Köllner et al., 2012
3.4 × 10^4^ – 4.2 × 10^7^ g^−1^ (wet)	Freshwater sediment	qPCR on GH18 genes	Xiao et al., 2005
Up to ~8.5 × 10^4^ g^−1^ (dry)	Freshwater sediment	qPCR on GH18 genes	Köllner et al., 2012
2.5 × 10^3^ g^−1^ (wet)	Soil	qPCR on GH18 genes	Xiao et al., 2005
2.3 × 10^8^ – 9.3 × 10^9^ g^−1^ (dry)	Soil	qPCR on GH18 genes	Gschwendtner et al., 2010
7 × 10^5^ – 9.3 × 10^6^ g^−1^ (wet)	Soil	qPCR on GH18 genes	Brankatschk et al., 2011
3 × 10^7^ g^−1^ (wet)	Soil	qPCR on GH18 genes	Kielak et al., 2013
^*^4.6 × 10^6^ – 1.1 × 10^7^ g^−1^ (wet)	Soil	qPCR on GH18 genes	Cretoiu et al., 2012

1 qPCR: quantitative polymerase chain reaction.

2 GH18: family 18 glycoside hydrolase.

* Values derived from digitalized figures using the Engauge Digitizer Program (http://digitizer.sourceforge.net/index.php?c=5).

Temperature is often considered as a critical factor controlling chitin degradation rates. There are several reports of variation in chitin degradation rates with the highest activity during periods of high *in situ* temperature (Hood and Meyers, 1977; Rodríguez-Kábana et al., 1983; Hillman et al., 1989; Gooday et al., 1991; Ueno et al., 1991; Boyer, 1994; Metcalfe et al., 2002). Analogously, observations that different chitinoclastic strains were isolated during different seasons provided support that temperature could also affect the composition of the chitinoclastic community (Warnes and Rux, 1982). In some of these studies reporting temperature dependency for chitin hydrolysis rates, substrate availability might have been a cryptic underlying factor driving the observed correlation. In aquatic ecosystems for example, chitinous zooplankton can be dominant contributors to polymeric chitin and are known to increase seasonally in response to warmer temperature. In agreement with this, Beier et al. (2012) recently detected pronounced seasonal dynamics in the chitinolytic community using cultivation-independent molecular methods, but it was not evident from this study if temperature or alternate autocorrelated environmental factors such as chitin supply via crustacean zooplankton were the major environmental factors driving the community shifts.

There are also studies that revealed that temperature seems to play a minor role: in the York River, the correlation between chitin degradation and temperature was much less evident in the water column compared to the sediments (Boyer, 1994). Further exceptions are reported for the North Sea where higher chitin degradation rates were observed in October/November compared to the warmer period during July/August (Gooday et al., 1991). Also in these studies, however, chitin availability seemed to have influenced chitin degradation rates, as maximum chitinase activity coincided with high abundances of chitin-containing organisms (Kirchman and White, 1999; LeCleir and Hollibaugh, 2006). In aquatic systems, the water-sediment interface represents a habitat where chitin accumulates as a result of sedimentation of chitinous particles. This environment is usually also identified as a hotspot for chitin degradation when compared to the water column or the bulk sediment (Hood and Meyers, 1977; Warnes and Rux, 1982; Gooday et al., 1991). In soils, decreasing chitinase activity has been observed over depth and, this pattern has been attributed to the higher presence of chitin-containing organisms in the upper soil layers (Rodríguez-Kábana et al., 1983; Ueno et al., 1991). A direct coupling of chitin concentration and the chitinolytic community has also been demonstrated in an experiment where chitin-amendment of a soil caused an increase in chitinase gene copy numbers (Xiao et al., 2005; Kielak et al., 2013).

Only a few studies have directly related measured hydrolysis rates to shifts on the chitinolytic community: It has for example been shown that high chitinase activity measured after a soil was amended with sludge or chitin was accompanied by a decrease in the diversity of chitinases (Metcalfe et al., 2002; Kielak et al., 2013). Two recent studies in a terrestrial and an aquatic environment also reported a significant correlation between chitinase gene copy numbers and measured chitin hydrolysis rates (Brankatschk et al., 2011; Köllner et al., 2012). A correlation between changes in the composition of the chitinolytic community and chitin hydrolysis rates has also been observed in a temporal survey of lake bacterioplankton (Beier et al., 2012), which indicates that apart from environmental factors also the community composition *per se* could be decisive for measured rates.

In summary, the available data suggest that temperature and chitin supply are important environmental factors controlling both chitin hydrolysis rates and the chitinolytic community structure. This further implies the existence of a link between dynamic shifts in the chitinolytic community and measured chitin hydrolysis rates across spatially or temporally connected habitats. Based on these observations, we speculate that organisms that contribute in significant ways to chitin degradation may in fact be specialized on chitin substrate use even if they likely also are able to metabolize other substrates.

Environments with limited connectivity or gene flow, such as systems located in different climate zones or systems that vary in salinity, have been shown to host dramatically different chitinolytic communities (Terahara et al., 2009; Beier et al., 2011; Manucharova et al., 2011). Recent evidence suggests that such isolated communities are adapted to the local prevailing conditions, as it was shown that the temperature optimum for maximal chitin degradation in soil was strongly correlated to the climate zone where the samples originated from (Manucharova et al., 2011). There may, however, still be constraints on such local adaptation, as suggested by Kang et al. (2005) who demonstrated a significant positive correlation of β-N-acetylhexosaminidase activities in wetlands with the annual mean temperature of the respective system. Future molecular studies targeting expression patterns for chitinases coupled to the presence of chitinase genes and measured rates would no doubt greatly increase our ability to decipher the mechanisms and controls underlying the process of chitin degradation, not least by identifying key players and their sensitivity to environmental change.

## Concluding remarks

In the previous sections, mechanisms and ecophysiological strategies of microbial chitin degradation and the role of parameters, such as temperature and chitin supply in determining chitin degradation rates have been discussed along with an account of compositional variation in chitinoclastic communities. The absence of long-term accumulation of chitin in natural systems implies that de novo production of chitin is the ultimate limiting factor controlling its degradation and turnover in nature. Still, the fate of this material with regards to production of new biomass or complete mineralization to inorganic constituents varies to a considerably and the underlying factors controlling this variation are only marginally understood. Besides the presence of other, more readily degraded substrates, also the composition of the bacterial community involved into chitin utilization could influence the fraction of chitin being mineralized, i.e., by the substrate affinity toward hydrolyses products. Habitat structure might determine such general characteristics of the inherent chitin utilizing community and therefore also dictate the fate of chitin in terms of its mineralization rates. This may have major implications for the cycling of carbon and nitrogen in food webs i.e., by carbon or nitrogen removal due to mineralization and volatilization. We therefore conclude that the interactive roles of habitat and the chitinolytic or chitin utilizing community and their taxonomic identification merits further investigation.

The process of chitin degradation is easier to target than degradation of many other polymers such as the structurally heterogeneous lignin and humic acids or even cellulose. This is because of its simple structure and the existence of primer-systems targeting the chitin modifying enzymes. Chitin degradation could therefore be explored as a general model for understanding microbial degradation of biopolymers in the biosphere.

### Conflict of interest statement

The authors declare that the research was conducted in the absence of any commercial or financial relationships that could be construed as a potential conflict of interest.
